# 30-Month Follow-Up of Individual Placement and Support (IPS) and Cognitive Remediation for People with Severe Mental Illness: Results from a Randomized Clinical Trial

**DOI:** 10.1155/2023/2789891

**Published:** 2023-04-28

**Authors:** Thomas Nordahl Christensen, Iben Gammelgård Wallstrøm, Elsebeth Stenager, Lone Hellström, Anders Bo Bojesen, Merete Nordentoft, Lene Falgaard Eplov

**Affiliations:** ^1^Copenhagen Research Centre for Mental Health, Mental Health Services, Copenhagen University Hospital, Copenhagen, Denmark; ^2^Research Unit of Psychiatry, Institute of Clinical Research, University of Southern Denmark, Odense, Denmark; ^3^Research Unit Mental Health, Children and Adult, Aabenraa, Department of Regional Health Research, University of Southern Denmark, Odense, Denmark; ^4^Department of Clinical Medicine, University of Copenhagen, Copenhagen, Denmark

## Abstract

**Background:**

The individual placement and support (IPS) model for persons with severe mental illness has proven to be more effective than traditional vocational approaches in improving competitive work over 18 months. In this study, the longer-term effects of IPS over 30 months were investigated in a Danish setting.

**Method:**

In a randomized clinical trial, we compared the effects of IPS, IPS enhanced with cognitive remediation and work-related social skills training (IPSE), and service as usual (SAU). At three locations in Denmark, 720 patients with serious mental illnesses were randomly assigned to the three groups. Competitive employment, education, and hospital admissions were tracked for 30 months using Danish national registers.

**Results:**

The beneficial effects of IPS on competitive employment and education at the 18-month follow-up were sustained over the 30-month follow-up period. Participants receiving IPS or IPSE were more likely to obtain competitive employment or education than those who received service as usual (IPS 65%, IPSE 65%, SAU 53%, *p* = 0.006), and they worked on average more weeks competitively (IPS 25 weeks, IPSE 21 weeks, SAU 17 weeks; IPS vs. SAU *p* = 0.004 and IPSE vs. SAU *p* = 0.007). Moreover, participants in the two IPS groups had fewer outpatient visits during the 30-month follow-up. However, this was only statistically significant when comparing IPSE with SAU *p* = 0.017.

**Conclusion:**

In conclusion, IPS and IPS enhanced with cognitive remediation and work-related skills training demonstrated that the vocational effects of the interventions are retrained over 30 months in a Danish context.

## 1. Introduction

Compared to alternative vocational rehabilitation programs, the individual placement and support (IPS) model has consistently shown superiority in randomized controlled trials [1]. IPS is a standardized supported employment approach comprising eight critical principles recognized as essential for success when supporting people with severe mental illness to gain and retain employment [2]. In short, IPS participants receive ongoing and individualized support for obtaining competitive employment or education where prolonged prevocational training is avoided. There is a strong focus on participants' job preferences, and the intervention is integrated into mental health services [2, 3].

In Denmark, the effects of IPS and IPS enhanced with cognitive remediation and work-related social skills training (IPSE) were tested in a randomized trial from 2012 to 2018. In this trial, the IPS and IPSE participants had higher employment and study rates and spent more time working than those enrolled in a standard vocational rehabilitation program (SAU). Moreover, IPS was cost-effective, and the participants in the two IPS groups obtained employment or education faster and were more satisfied compared with those receiving SAU [4–6]. The Danish trial is comparable to most previous IPS trials with regard to the follow-up period. Most IPS trials have follow-up periods of 18 months or less, and the evidence of long-term effectiveness beyond 24 months is less established [7]. Nonetheless, there is some evidence of the long-term effect of IPS internationally. IPS trials from Hong Kong [8], Switzerland [9], and the US [10] with 3- and 5-year follow-up periods showed favorable effects of IPS compared with traditional vocational rehabilitation. Moreover, two small-uncontrolled studies from the US with 8 to 12-year follow-ups showed that 71% worked for more than half of the follow-up years [11] and that 75% of the participants worked beyond the initial study period, with 33% working at least five years during the ten years [12]. However, these studies are limited by relatively small sample sizes, lack of control conditions, and may have problems with recall bias because the outcomes were dependent on whether the participants can recall many years of work history.

Thus, there is strong evidence that IPS effectively improves the competitive work outcomes of people with severe mental illness, but its longer-term impact is less clear. The overall aim of the present study was to investigate if the effects of IPS and IPS enhanced with cognitive remediation and work-related social skills training (IPSE) found at 18-month follow-up are maintained over 30 months using data from Danish national registers.

## 2. Method

The effects of IPS, IPSE, and SAU were investigated in a randomized, three-arm parallel, multisite, superiority trial with blinded outcome assessment. Trial protocol [4] was conducted before the RCT and registered at ClinicalTrials.gov, NCT01722344. Moreover, the trial was approved by the Ethics Committee in the Capital Region of Denmark (registration #H-3-2012FSP34) and the Danish Data Protection Agency (registration #01768 RHP-2012-011).

The addition of a second site (Silkeborg) and the division of the Copenhagen team into two independent teams were the only modifications made to the original trial design, which enabled the recruitment of a sufficient number of participants in accordance with the sample size estimation.

### 2.1. Participants and Recruitment

The participants were either recruited by the psychiatric case managers or by themselves. Before randomization, a qualified and trained researcher assessed participants in a three-hour interview. The diagnostic interview tool schedules for clinical assessment in neuropsychiatry (SCAN) was used to evaluate if participants met the diagnostic criteria. Participants were eligible for the trial if they had a diagnosis of schizophrenia, schizotypal, or delusional disorders (F20–F29, ICD10), a diagnosis of bipolar disorder (F31, ICD10), or a diagnosis of recurrent depression (F33, ICD10). They should reside in one of three Danish cities: Copenhagen/Frederiksberg, Odense, or Silkeborg, and be assigned to early intervention teams (OPUS teams) or community mental health services. All participants should be unemployed and not in education at baseline, but they should express a clear desire for competitive employment or education.

Moreover, they should be able to speak and understand Danish sufficiently well to participate without an interpreter and be between 18 and 64 years old. The only exclusion criteria were if the participant refused to give informed consent or received a retirement pension. According to the zero exclusion criteria in IPS, no patients were excluded due to poor work history, low function, severe symptoms, or substance abuse.

### 2.2. Randomization and Blinding

Eligible participants were randomly assigned to IPS, IPSE, or SAU after the baseline assessment. The randomization was computer-generated with a random allocation sequence and varying block sizes and stratified by site, sex, work history, and work readiness. The assessors and the research team were blinded to participants' allocation, and the randomization code was not revealed before all analyses at the 18-month follow-up were performed, and the conclusion was drawn. Hence, for this 30-month follow-up, the research team could not be blinded. It was accepted that participants, employment specialists, and the mental health team were unblinded to the allocation and, consequently, the risk of bias that may arise with this decision.

## 3. Interventions

Regardless of group allocation, all participants received the same level of psychiatric care from early intervention or community mental health teams [13]. The psychiatric treatment consisted of at least individual case management based on cognitive therapeutic methods and medical review.

### 3.1. Individual Placement and Support (IPS)

Participants allocated to the IPS group received a service that complied with the eight key principles of IPS. The IPS manual [3], including worksheets, was translated into Danish before the trial. The employment specialists, who had a caseload of maximum of 25 participants, were recruited from the national Danish job centers, and they were part of a team consisting of at least one IPS team leader and three employment specialists. The IPS teams were integrated into the outpatient mental health teams, and the employment specialists had individual meetings with case managers and participated in medical conferences. Most of the working week was devoted to contacting potential employers and supporting participants in applying for and obtaining competitive employment or education based on the participants' preferences. Once the participants were employed or had started education, follow-along support was provided. The intervention was time-unlimited and continued as long as the participant wanted and needed support. The participants were enrolled for 12 months on average. Competitive employment was defined as part-time or full-time work open to all persons and remunerated by at least the minimum wage for hours worked. Education was related to an employment goal and was not designed specifically for people with disabilities.

### 3.2. IPS Enhanced with Cognitive Remediation and Work-Related Social Skills Training (IPSE)

The cognitive remediation consisted of cognitive computer training using the software CIRCuiTS [14] and group-based training in cognitive coping strategies following the thinking skills for work program and extended with work-related social skills training (WSST) [15, 16]. The computer training consisted of computer exercises that target strategy use and cognitive functions such as attention, memory, and executive functioning. Before the computer exercises, participants defined goals and identified strategies to improve their cognitive performance, which was regularly reviewed and modified [14]. The tasks gradually increased in difficulty depending on individual performance. In addition to the computer training, the program included teaching coping and compensatory strategies and providing support to generalize to everyday activities, such as using a calendar to improve planning abilities and developing routines to compensate for persisting difficulties and optimize work functioning.

Moreover, six sessions of work-related social skills training were offered. The aim was to develop, train, maintain, and generalize communication skills essential in achieving or retaining jobs or education and to train basic emotional and cognitive skills. Attention was paid to problem-solving skills and conflict management, as well as training in decoding norms for social interaction and how to be better at making informed and well-considered decisions. The intervention was performed in groups with a maximum of eight participants, and the sessions included an introduction of concepts, role-plays, homework exercises, and a review of real-world successes and failures. The group sessions were led by trained psychologists, and employment specialists participated as cotherapists. The sessions were offered twice a week in 90-minute sessions. In total, 24 sessions with computer training and the teaching of coping strategies and an additional six sessions with social skills training were offered.

### 3.3. Control Group (SAU)

Control group participants continued to receive psychiatric outpatient treatment and counseling at the job centers. Based on the register data, the participants were referred to various vocational support options provided by the job centers and private companies from baseline to the 18-month follow-up. This consisted of meetings at the job center (mean = four meetings), mentor support (mean = 27 h), skills training courses, unpaid internships, and transitional employment (384 hours).

## 4. Outcome Measures

All included outcomes in the present study are considered exploratory outcomes because the primary and secondary outcomes of the trial were reported with 18-month follow-up data. The first outcome is the difference in weeks between groups in competitive employment or education measured from baseline until a 30-month follow-up using the Danish Register for Evaluation of Marginalization (DREAM) database [17, 18]. The register covers the entire population and contains employment data, including salaries and education. The same outcome was divided into weeks of competitive employment or weeks of education. The second outcome was employment or education at one point during the follow-up period. The third outcome was the difference between groups in time to employment or education. The fourth outcome was differences between groups in psychiatric outpatient visits and hospitalization extracted from the Danish national patient registry [19]. In addition, three unpublished nonvocational survey outcomes from the 18-month follow-up are reported. This includes differences between groups in health-related quality of life measured with the 12-item short form health survey (SF-12) [20], social functioning measured with the global assessment of functioning (GAF-F) [21], and empowerment assessed by the empowerment scale (ES) [22].

## 5. Statistical Analysis

All analysis was based on the intention to treat principles, and because all the data included were register-based, we had a complete follow-up on all outcomes. We report baseline characteristics with mean and standard deviations (SD) for numeric variables and for categorical variables count (*n*) with percentages.

All estimates except the survival analysis are reported with a success rate difference (SRD) [23] with bootstrapped inferential statistics, which was the same method used when the 18-month results were reported. For dichotomous outcomes, the SRD is simply the difference between the proportion of vocational success in two groups. For numerical outcomes, the SRD is derived from Wilcoxon's *U* statistic:  *SRD* = 2*U*/(*N*_0_∙*N*_1_) − 1, where *U* is the Wilcoxon's *U* statistic and *N*_0_ and *N*_1_ are the sample sizes for the two groups. For numerical outcomes, this amounts to the difference in the probability of a random patient in the intervention group having a better outcome than a random patient in the comparison group. The probability of a random patient in the comparison group scoring higher than a random patient in the intervention group. Scores above 0, therefore, implicate a higher numerical value for the IPS group compared with the SAU group. Scores below 0 indicate a higher numerical value in the SAU group. Days to employment or education were analyzed using the Cox proportional hazard regression and reported using hazard ratios and Kaplan-Meier survival curves. All outcomes on outpatient visits and hospitalization are reported with mean and median with standard deviation (SD) and interquartile range (IqR), and *p* values are derived from the Wilcoxon statistical test. Because we compare all three groups, the *p* values for the outcomes should be interpreted according to an adjustment of the alpha level to a third (.05/3 = .0167), and 98.3% CI are reported for all effect estimates.

## 6. Results

**Figure 1:**
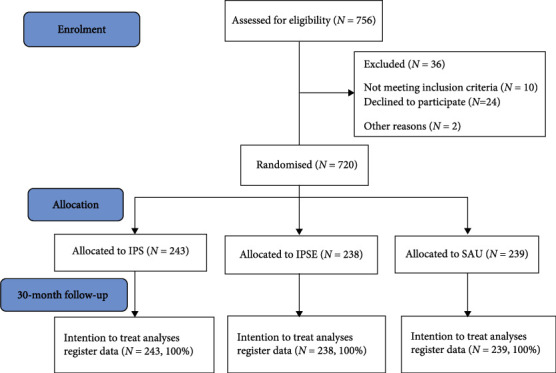
Study flow-chart.

Of the 756 participants assessed for eligibility, 36 were excluded: 10 did not meet the inclusion criteria, 24 declined to participate after they received more information about the trial, and two moved away before the baseline interview. A total of 720 participants were randomly assigned to the three groups: (1) IPS (*N* = 243), (2) IPSE (*N* = 238), and (3) SAU (*N* = 239). Because all measures in this study were register-based, there was a 100% follow-up (Figure 1).

At baseline, the participants' average age was 33 (SD 9.9) years, and 48% of them were women. The majority (77%) had a schizophrenia spectrum illness, whereas the remaining participants (12% and 11%, respectively) had a bipolar affective disorder or recurrent depression. The participants had a generally low level of education, with 39% having only completed elementary or lower secondary school. Moreover, the participants' global level of cognitive functioning was -2.70 standard deviations lower compared with the reference healthy population, measured on the Brief Assessment of Cognition in Schizophrenia (BACS) scale (Table 1). The mean IPS fidelity score of each site ranged from 75 to 101, measured on the IPS-25 scale, which indicates fair or good levels of IPS fidelity and adherence to the eight key principles of the IPS model.

In the 30-month follow-up period, IPS participants were more likely to work competitively or be enrolled in education at one point than those in the SAU group (65% vs. 52.7%; SRD, 0.123 [98% CI 0.012-0.231]; *p* = 0.006). Similar significant results were found when comparing IPSE with SAU (65.1% vs. 52.7%; SRD, 0.124 [98.3% CI 0.015-0.233]; *p* = 0.006). Additionally, there was a statistically significant difference between IPS and SAU when analyzing only competitive employment (46.5% vs. 35.1%; SRD, 0.114 [98.3% CI 0.006-0.226] *p* = 0.011), but no difference was found between IPSE and SAU (41.6% vs. 35.1%; SRD, 0.065 [98% CI -0.036–0.174]; *p* = 0.148). Over the 30-month follow-up period, there was also a significant difference of 9.2 weeks in competitive employment or education between IPS and SAU giving an SRD of 0.146 (98% CI 0.02-0.268), *p* = 0,004. The difference between IPSE and SAU was 8.2 weeks, which gave an SRD of 0.139 (98% CI 0.022-0.265), *p* = 0.007. When analyzing only competitive employment, there was a difference between IPS and SAU of 7.6 weeks, giving an SRD of 0.126 (98.3% CI 0.012-0.243) *p* = 0.007, but no differences were found between IPSE and SAU in this outcome (SRD, 0.069 [98.3% CI -0.046–0.177]; *p* = 0.134). For a full overview, see Table 2.

**Figure 2:**
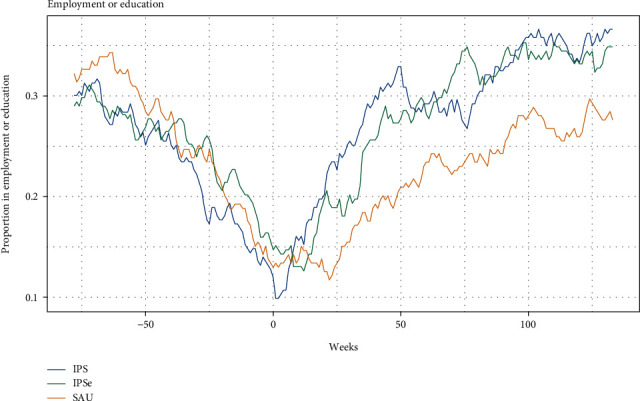
Employment and education rates at any given week from 75 weeks before baseline to 141 weeks after baseline.

**Figure 3:**
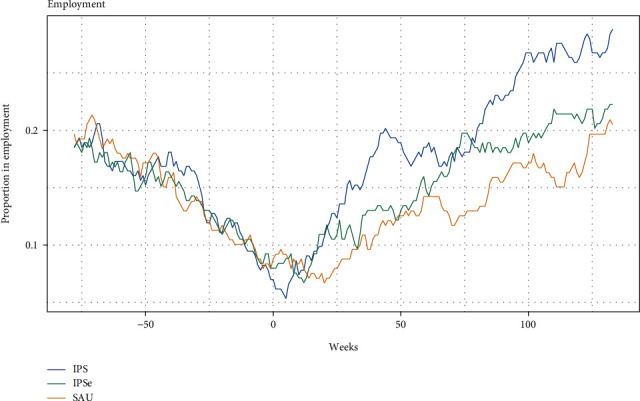
Employment rates at any given week from 75 weeks before baseline to 141 weeks after baseline.

Figures 2 and 3 illustrate the difference between the groups in employment or education at any given week in the 30-month follow-up period. We found that the participants in the two IPS groups from week 12 after baseline to the 30-month follow-up were holding more competitive jobs and education on average when compared to SAU. In the last week of the 30 months, 38% of the IPS group were in education or competitively employed. This was the case for 35% of the IPSE group and 27% of the SAU group. When analyzing competitive employment separately, there was an increase in the employment rates in all three groups during the 30 months, but in the last weeks of the period, the IPSE and SAU groups were less employed, with 21% and 22%, respectively, compared with 28% in the IPS group.

**Figure 4:**
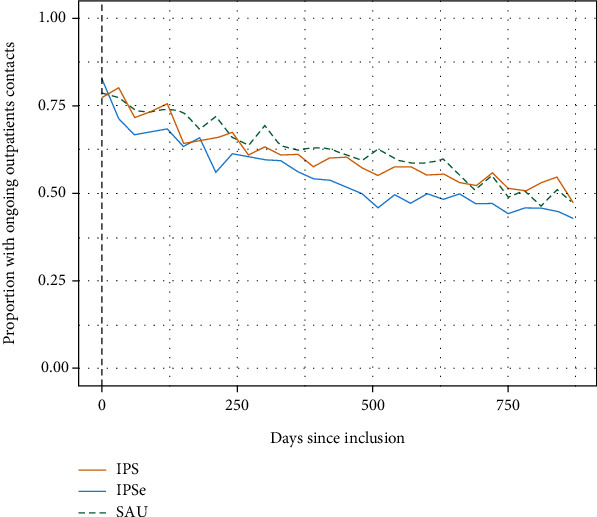
The average outpatient contacts per month over the 30-month follow-up.

When analyzing time to employment and education using Cox regression, a significant difference between IPS vs. SAU was found (hazard ratio, 1.52 [98.3% CI, 1.09-2.10]; *p* = 0.002). When only competitive employment was analyzed, there was a statistically significant difference between IPS and SAU (Table 3). The online supplementary provides the Kaplan-Meier curves. As shown in Table 4, we found a difference between the groups in the use of psychiatric care, where the participants in the two IPS groups had fewer psychiatric outpatient contacts. IPS participants had, on average, 49, IPSE 48, and SAU 55 contacts. However, when we tested for this difference, the results were only statistically significant when comparing IPSE with SAU. Overall, the two IPS groups had fewer days hospitalized when compared with SAU, but this difference was not statistically significant (Table 4).  Figure 4 shows the average outpatient contacts per month over the 30-month follow-up period. No differences were found between the three groups in health-related quality of life (SF-12), social functioning (GAF-F), or empowerment (ES) at the 18-month follow-up (online Supplementary (available here)).

## 7. Discussion

This study's key finding was that participants in the IPS or IPSE interventions for people with serious mental illnesses had more weeks in competitive employment or education than those who participated in traditional vocational rehabilitation. IPS participants worked more weeks in competitive employment compared with SAU, but this was not the case for IPSE participants. Moreover, the IPS groups had fewer psychiatric outpatient visits when compared with SAU.

The findings suggest that the beneficial vocational effects of IPS are sustained over the 30-month follow-up but also that the supplement to IPS with cognitive remediation and work-related social skills training does not add any additional effects. There was no difference between the IPSE and SAU when not including education in the outcome measure. This finding was similar to the results from the 18-month follow-up, and as previously reported, the lack of additional effect may be explained by a relatively high dropout rate. 24% of the IPSE participants did not attend any sessions with cognitive remediation or work-related social skills training due to a lack of motivation or fear of participating in a group setting. Moreover, only 52% of the participants attended more than 6 out of the 30 sessions.

The present trial was different from most prior IPS trials in that we also included participants who, at baseline, intended to pursue education rather than employment. As a result, it is challenging to draw comparisons with prior international IPS trials that only included participants who intended to pursue employment. However, Hoffmann et al. found in a 5-year follow-up of an IPS trial from Switzerland that 65% in the IPS group obtained employment, and they worked on average 107 weeks compared with 37 weeks in the SAU group [9]. In comparison, there were 46.5% in the Danish IPS group who obtained competitive employment, and when education was added to the measure, 65% obtained employment or education over a 2.5-year follow-up. When looking at weeks of employment in the present trial, the IPS group, on average, worked 25 weeks compared with 17 weeks in the SAU group. One explanation for why we are not finding the same rates of competitive employment as reported in previous IPS trials may be that about half of the participants had education as their primary goal rather than employment, and the follow-up period was insufficient to confirm that the education later transferred into competitive employment.

Interestingly, receiving IPS service in the present study was also associated with lower levels of outpatient psychiatric treatment over the 30 months. Most randomized trials of IPS have not reported such differences in psychiatric care besides a European multisite study [24] and a trial from Switzerland [9]. The substantial integration of IPS within local mental health care may help to explain the difference in outpatient contacts reported in the present trial. The psychiatric case managers revealed that they spent less time on social work and used less time on meetings with the personnel at the job centers after IPS started. Because the patients' social benefits counseling and support for obtaining and maintaining a job or education were provided by the IPS employment specialists, it is possible that they had less need to engage with the psychiatric case managers.

### 7.1. Strengths and limitations

The use of representative longitudinal register data for the complete population with 100% follow-up, providing accurate information on vocational results, and use of psychiatric care was the study's main strength.

Also, randomization stratifying for important predictive factors and a large sample of 720 participants increased the quality of the trial. Moreover, fidelity ratings were performed throughout the entire trial period to ensure adherence to the IPS method.

A limitation in the trial was that the participants, employment specialists, and mental health care staff were unblinded to the participants' allocation, and in this 30-month follow-up, the researchers were also unblinded. Moreover, as reported earlier, the length and number of cognitive and social skills training sessions may not have been sufficient to archive strong effect sizes for the IPSE group due to a high dropout rate.

## 8. Implication

Based on the findings of this trial, we propose that all municipalities in Denmark apply the IPS strategy to increase the employment rates of people with severe mental illness. Since the results from the 18-month follow-up, an increasing number of municipalities in Denmark have decided to implement the IPS model in the job centers. The results from this 30-month follow-up study add another good argument for making a national strategy in Denmark where all citizens with serious mental illnesses are offered evidence-based IPS service. However, there is still a need for further research with longer follow-up periods to determine if the obtained education is completed and further transferred into competitive employment. As we did not find any significant difference between IPS and IPS enhanced with cognitive remediation and social skills training, it is still unclear if this enhancement adds additional effects to the IPS intervention.

## Figures and Tables

**Table 1:** Baseline characteristics of 720 participants in the trial randomized to IPS, IPSE, or SAU.

	IPS (*N* = 243)	IPSE (*N* = 238)	SAU (*N* = 239)
Sex, *N* (%)			
Female	94 (38.7)	87 (36.6)	95 (39.8)
Male	149 (61.3)	151 (63.5)	144 (60.3)
Age, mean (SD)	33.3 (10.3)	33.0 (9.5)	32.8 (9.9)
Previous work history *N* (%)^∗^			
No	125 (51.4)	117 (49.2)	123 (51.5)
Yes	118 (48.6)	121 (50.8)	116 (48.5)
Education, *N* (%)			
Master or equivalent	13 (5.4)	14 (5.9)	21 (8.8)
Bachelor or equivalent	28 (11.5)	22 (9.2)	28 (11.7)
Short-term tertiary education	43 (17.7)	53 (22.3)	44 (18.4)
Upper secondary education	61 (25.1)	57 (24.0)	57 (23.9)
Primary/lower secondary education	98 (40.3)	92 (38.7)	89 (37.2)
Married or cohabiting, *N* (%)			
No	197 (81.1)	194 (81.5)	187 (78.2)
Yes	46 (18.9)	44 (18.5)	52 (21.8)
Site, *N* (%)			
Copenhagen, Frederiksberg	174 (71.6)	165 (69.3)	169 (70.7)
Odense, Silkeborg	69 (28.4)	73 (30.7)	70 (29.3)
Diagnoses, *N* (%)			
Schizophrenia spectrum disorders (ICD10 codes: F20-F29), *N* (%)	184 (75.7)	181 (76.1)	186 (77.8)
Bipolar disorder (ICD10 codes: F31.0-F31.9), *N* (%)	32 (13.2)	30 (12.6)	25 (10.5)
Recurrent depression (ICD10 codes: F33.0-F33.9), *N* (%)	27 (11.1)	27 (11.3)	28 (11.7)
PSP score, mean (SD)	47.3 (10.8)	47.2 (10.8)	47.0 (10.0)
Psychotic symptoms (SAPS), mean (SD)	1.2 (1.3)	1.2 (1.3)	1.2 (1.3)
Negative symptoms (SANS), mean (SD)	1.9 (0.8)	1.9 (0.8)	2.0 (0.8)
Disorganized symptoms (SAPS/SANS), mean (SD)	0.3 (0.5)	0.3 (0.5)	0.3 (0.5)
BACS global, mean (SD)	-2.6 (1.61)	-2.8 (1.9)	-2.7 (1.8)
Hamilton score, mean (SD)	6.0 (4.2)	6.4 (4.2)	6.8 (4.1)
Self-efficacy, mean (SD)	14.1 (6.3)	14.3 (6.1)	13.1 (6.4)
Rosenberg's self-esteem (SD)	15.6 (6.1)	15.6 (5.7)	16.0 (5.9)
SF-12 total (SD)	83.4 (7.9)	82.0 (7.9)	81.5 (7.8)

^∗^Previous work history: ≥2 months paid job last five years.

**Table 2:** Comparison of effect on exploratory vocational outcomes after 30 months of follow-up for 720 patients with severe mental illness randomized to IPS, IPSE, and SAU.

Outcomes	IPS	IPSE	SAU	IPS vs. SAU		IPSE vs. SAU		IPS vs. IPSE	
Weeks in labor force engagement	Mean (SD)	Mean (SD)	Mean (SD)	Success rate difference (98.3% CI)	*p* value (Boot. *p* value)	Success rate difference (98.3% CI)	*p* value (Boot. *p* value)	Success rate difference (98.3% CI)	*p* value (Boot. *p* value)
Weeks in employment and education	37.8 (41.3)	36.8 (40.1)	28.6 (39.4)	0.1460.02-0.268	0.004 (0.005)	0.139 (0.022-0.256)	0.007 (0.008)	0.009 (-0.116-0.130)	0.863 (0.864)
Weeks in employment	24.9 (37.1)	20.6 (33.5)	17.3 (32.9)	0.126 (0.012-0.243)	0.007 (0.006)	0.069 (-0.046-0.177)	0.134 (0.135)	0.059 (-0.061-0.170)	0.217 (0.214)
Weeks in education	14.0 (27.5)	17.2 (30.8)	12.0 (27.1)	0.042 (-0.067-0.142)	0.332 (0.329)	0.08 (-0.025-0.181)	0.07 (0.071)	-0.039 (-0.146-0.060)	0.385 (0.386)
Labor force engagement at any point	%	%	%	Success rate difference (98.3% CI)	*p* value (imp. based	Success rate difference (98.3% CI)	*p* value (imp. based)	Success rate difference (98.3% CI)	*p* value (imp. based)
Employment or education at any point	65.0%	65.1%	52.7%	0.123 (0.012-0.231)	0.006 (0.008)	0.124 (0.015-0.233)	0.006 (0.007)	-0.001 (-0.101-0.104)	0.981 (0.988)
Employment at any point	46.5%	41.6%	35.1%	0.114 (0.006-0.226)	0.011 (0.016)	0.065 (-0.036-0.174)	0.148 (0.148)	0.049 (-0.058-0.157)	0.279 (0.279)
Education at any point	32.5%	35.3%	28.9%	0.036 (-0.068-0.132)	0.387 (0.391)	0.064 (-0.036-0.169)	0.133 (0.129)	-0.028 (-0.132-0.073)	0.520 (0.513)

**Table 3:** Days to employment and education for 720 patients with severe mental illness randomized to IPS, IPSE, and SAU.

	IPS vs. SAU		IPSE vs. SAU		IPS vs. IPSE	
	HR (98.3% CI)	*p* value	HR (98.3% CI)	*p* value	HR (98.3% CI)	*p* value
Employment or education	1.515 (1.09-2.10)	0.002	1.364 (0.98-1.90)	0.026	1.114 (0.82-1.51)	0.398
Employment	1.564 (1.06-2.30)	0.005	1.254 (0.84-1.87)	0.176	1.251 (0.87-1.80)	0.142
Education	1.202 (0.78-1.87)	0.314	1.242 (0.80-1.92)	0.236	0.972 (0.64-1.48)	0.870

**Table 4:** Comparison of the use of psychiatry in the 30 months of follow-up for 720 patients with severe mental illness randomized to IPS, IPSE, and SAU.

Outcomes	IPS		IPSE		SAU		IPS vs. SAU	IPSE vs. SAU	IPSE vs. IPS
	Mean (SD)	Median (IqR)	Mean (SD)	Median (IqR)	Mean (SD)	Median (IqR)	*p* values	*p* values	*p* values
Outpatient visits	48.8 (40.1)	43.0 (16.5-68.5)	47.8 (48.3)	33.5 (15.0-62.5)	55.4 (49.5)	41.0 (22.0-74.0)	0.240	0.017	0.268
Outpatient course	2.8 (3.3)	2.0 (1.0-3.0)	2.5 (2.7)	2.0 (1.0-3.0)	2.9 (3.3)	2.0 (1.0-4.0)	0.809	0.258	0.388
Emergency visits	0.15 (0.91)	0.00 (0.00-0.00)	0.21 (0.83)	0.00 (0.00-0.00)	0.10 (0.36)	0.00 (0.00-0.00)	0.297	0.363	0.057
Hospitalisations	1.3 (2.9)	0.0 (0.0-1.0)	1.0 (2.3)	0.0 (0.0-1.0)	1.3 (2.8)	0.0 (0.0-1.0)	0.242	0.269	0.886
Days hospitalized	21.9 (62.2)	0.0 (0.0-10.0)	18.9 (50.4)	0.0 (0.0-5.0)	30.9 (85.6)	0.0 (0.0-12.5)	0.313	0.336	0.895
	*N* (%)		*N* (%)		*N* (%)		*p* values	*p* values	*p* values
Outpatient visits	242 (99.6)		233 (97.9)		234 (97.9)		0.097	0.996	0.096
Emergency visits	13 (5.3)		24 (10.1)		19 (7.9)		0.252	0.417	0.052
Hospitalisations	76 (31.3)		80 (33.6)		89 (37.2)		0.168	0.409	0.585

## Data Availability

Due to the general data protection regulation, the register-based data used in this study cannot be publicly available. The data used is provided and hosted by Statistics Denmark, and only the authors of this study are allowed access.

## Supplementary Materials

Supplementary Materials Figure E1: the Kaplan-Meier curves of time to employment or education. Table 1: comparison of the nonvocational effect after 18 months of follow-up for 720 patients with severe mental illness randomized to the 3 study groups.
