# Supplementary material for: 30-Month Follow-Up of Individual Placement and Support (IPS) and Cognitive Remediation for People with Severe Mental Illness: Results from a Randomized Clinical Trial
Source: Psychiatry J. 2023 Apr 28;2023:2789891. doi: 10.1155/2023/2789891 (PMC10162865; doi:10.1155/2023/2789891)
Supplement: Supplementary Materials — Figure E1: the Kaplan-Meier curves of time to employment or education. Table 1: comparison of the nonvocational effect after 18 months of follow-up for 720 patients with severe mental illness randomized to the 3 study groups. [file 2789891.f1.docx]

**Supplementary**

Figure E1 Time to employment or education

Tabel E1 Comparison of nonvocational effect after 18 Months’ Follow-up for 720 Patients with Severe Mental Illness Randomized to the 3 Study Groups

|  | IPS | IPS-e | SAU | IPS vs. SAU | | IPS vs. IPS-e | | IPS-e vs. SAU | |
| --- | --- | --- | --- | --- | --- | --- | --- | --- | --- |
|  | **Mean SD** | **Mean SD** | **Mean SD** | **Est. mean diff. CI** | **P-value imputation based** | **Est. mean diff. CI** | **P-value imputation based** | **Est. mean diff. CI** | **P-value imputation based** |
| GAF-D (0-100) | 48.6  12.4 | 50.9  13.8 | 47.7  11.8 | 0.16  -2.37,2.69 | 0.902  0.925 | -3.00  -5.67,-0.32 | 0.028  0.107 | 3.47  0.73,6.21 | 0.013  0.097 |
| Empowerment (ES) (0-84) | 49.9  8.5 | 50.3  8.8 | 49.0  9.6 | 0.86  -1.02,2.74 | 0.372  0.654 | -0.27  -2.14,1.60 | 0.775  0.337 | 1.12  -0.86,3.11 | 0.267  0.165 |
| SF12 Total | 83.8  7.2 | 83.2  8.0 | 83.6  8.7 | -0.33  -2.00,1.35 | 0.704  0.612 | -0.11  -1.75,1.53 | 0.894  0.883 | 0.01  -1.82,1.83 | 0.992  0.524 |
| SF12 Physical component | 38.2  5.2 | 37.3  5.8 | 38.7  5.8 | -0.46  -1.68,0.76 | 0.461  0.320 | 0.97  -0.26,2.20 | 0.123  0.233 | -1.35  -2.65,-0.05 | 0.043  0.031 |
| SF12 Mental component | 45.5  7.7 | 45.9  8.5 | 44.8  8.9 | 0.13  -1.60,1.85 | 0.886  0.850 | -1.09  -2.83,0.66 | 0.222  0.500 | 1.36  -0.52,3.23 | 0.157  0.383 |
